# Co-option and innovation in neural crest evolution

**DOI:** 10.1126/sciadv.aed9037

**Published:** 2026-06-24

**Authors:** Igor Adameyko

**Affiliations:** ^1^ Department of Neuroimmunology, Center for Brain Research, Medical University Vienna, 1090 Vienna, Austria.; ^2^ Department of Physiology and Pharmacology, Karolinska Institutet, 17177 Stockholm, Sweden.

## Abstract

The neural crest is one of the most evolutionarily transformative cell lineages in vertebrates. Defined by its migratory capacity and multipotency, it is not a fixed cell type but a transient, shape-shifting stem-like population that redistributes and repurposes diverse cellular programs after germ layer formation. In this opinion piece, we pursue three goals. First, we explore how the neural crest acts as an evolutionary opportunist and a thief, co-opting identities from mesodermal, placodal, and central nervous system lineages to generate novel structures. Second, we distinguish between such co-opted features and true innovations that arise within the permissive neural crest context. Third, we discuss an evolutionary scenario in which the neural crest originated from a photosensory and pigmented cell lineage involved in camouflage and light sensing. During the transition to predation, this ancestral nonessential and, therefore, plastic lineage acquired multipotency and migratory behavior, enabling the emergence of new cell types and contributing to major vertebrate innovations such as the “New Head.”

## Teaser

Neural crest evolution reassembled ancient programs in pigment cell lineage, resulting in vertebrate innovations.

## INTRODUCTION

The neural crest is a vertebrate innovation, a transient, multipotent, and migratory embryonic cell population that contributes to a substantial range of derivatives—craniofacial cartilage and bone, peripheral neurons and glia, pigment cells, and more (*1*, *2*). The origin of this lineage represents one of the most profound questions in evolutionary developmental biology (*3*–*11*). What evolutionary transitions led to the emergence of such a flexible and multifunctional embryonic cell type, which bridges the competence of germ layers? Also, critically, which developmental or physiological pressures may have driven its emergence?

According to the already classic knowledge, the neural crest develops from the neural plate border region, a zone of competence at the interface of the epidermis and neuroectoderm (*12*, *13*). Here, conserved patterning genes create a regulatory domain that, in vertebrates, becomes the launching point for neural crest emergence (*2*, *6*). However, while the border zone exists in amphioxus and tunicates (*14*, *15*), only vertebrates display the full migratory and multipotent neural crest phenotype, suggesting that new combinations of upstream regulators, such as FoxD3 (forkhead box D3), Ets1, Tfap2a/b (transcription factor AP-2 α/β), Msx1 (Msh homeobox 1), Sox10 [SRY (sex-determining region Y) box 10], Snai1/2 (snail family transcriptional repressor 1/2), and others (*2*), were required for the full neural crest module to become operational. This model accounts for the modular and layered assembly of the neural crest gene regulatory network but does not specify which selective pressures or ancestral functions might have scaffolded the earliest or even later transitions.

As an additional layer to this view, a more nuanced perspective sees the neural crest not simply as a novel invention but as an evolutionary hybrid—a lineage assembled through the fusion of multiple partial regulatory programs drawn from different germ layer territories (*6*, *16*). In this context, the work of Weston and colleagues (*17*–*20*) deserves particular mention, as they articulated a developmental distinction between the mesoderm-like, skeletogenic neural crest ectomesenchyme (which they termed “metablast”) and the neural crest that give rise to the peripheral nervous system. The hypothesis of the evolutionary hybrid is supported by the observation that neural crest cells recapitulate derivatives typical of both ectoderm and mesoderm: for example, pigment cells and peripheral neurons on the one hand and perivascular cells, dermis, bone, and cartilage on the other. Thus, the innovation of the neural crest could have involved the gradual integration of epithelial-to-mesenchymal transition (EMT) programs [Snail, Twist, and cadherin repression (*21*)] with sensory gene modules [Pou4f1 (POU class 4 homeobox 1; Brn3a) and Isl1 (*22*)] and pigment cell regulators [Mitf (melanocyte-inducing transcription factor), Sox10, and Pax3 (paired box 3) (*23*)] into a single, highly versatile lineage, which eventually expanded its developmental potential even further toward the autonomic nervous system and a plethora of mesodermal cell types. Overall, these and other views [(*3*, *4*); (*6*), chapters 1 to 6] aim to explain how genetic programs and cell fates were reshaped during neural crest evolution, and they collectively suggest a gradual process in which new cell fates were progressively added to the neural crest’s multipotent repertoire.

However, the major challenge is to develop a coherent reasoning about evolutionary pressures, lifestyle scenarios, and molecular solutions, together leading to the starting point for the very origin of protoneural crest. Thus, here, we would like to set up the main question of this review: What was before the neural crest and how did the neural crest originate under specific and currently unknown selective pressures and adaptive strategies.

On the basis of the current understanding of fates co-opted throughout evolution, we argue that the neural crest originated as a nonessential and safe evolutionary space derived from the lineage of former pigmented photoreceptors [(*6*), chapter 5; (*8*, *24*)], where developmental innovation occurred with reduced constraint, being free from the tight selective pressures imposed on the canonical germ layers and preexisting body plans. In doing so, the protoneural crest enabled a form of evolutionary plasticity that facilitated the origin of new traits through the modular reuse and recombination of preexisting regulatory developmental networks (*25*, *26*). All of that occurred during a marked transition from filter-feeding to active hunting lifestyle (*27*). Thus, the neural crest might be better understood in a broader way—not simply as a migratory multipotent cell type but as a cell lineage capable of integrating multiple unrelated regulatory modules and deploying them in new anatomical and physiological contexts.

## MESODERMAL MIMICRY BY AN ECTODERMAL LINEAGE—REVISITING THE NEW HEAD HYPOTHESIS

During vertebrate head development, cranial neural crest cells migrate into the pharyngeal arches and facial prominences, giving rise to bone, cartilage, perivascular cells, soft connective tissue (dermal fibroblasts, tenocytes, adipocytes, and smooth muscle cells)—components historically attributed to mesoderm (*28*, *29*). This skeletogenic potential of the neural crest mirrors that of the cranial paraxial mesoderm, a more ancient population that contributes to the same types of tissues in more posterior or axial regions (*30*). What is evolutionarily important is that while both cranial mesoderm and cranial neural crest can give rise to cartilage and bone, only the mesoderm retains the capacity to generate skeletal muscle and endothelial cells—lineages that are not produced by the neural crest cells. This key difference delineates the boundary of the neural crest’s mesodermal mimicry. This mesoderm-like behavior is facilitated by the activation of core mesenchymal transcriptional programs, which are also used in mesodermal skeletal development (*31*). Neural crest–derived ectomesenchyme essentially duplicates the mesodermal skeletogenic circuitry and executes it downstream of a different upstream neural crest–specific gene regulatory network (*2*, *31*, *32*). From an evolutionary perspective, this arrangement may have allowed the cranial neural crest to elaborate novel head morphologies without the need to invent new terminal differentiation pathways instead of rerouting preexisting mesodermal programs into a new migratory lineage. This capacity to generate anterior facial mesenchyme has often been linked—sometimes rather simplistically in more peripheral fields—to the emergence of articulated jaws and the modern vertebrate skull. The New Head Hypothesis (*27*), a highly influential vision of evolving cranial neural crest through the origin of anterior cranial mesoderm–like mesenchyme, is periodically misrepresented in superficial discussions as being primarily about late-arising features such as articulated jaws. In reality, Gans and Northcutt (*27*, *33*–*35*) originally framed their idea around an earlier transition: from a protochordate-like, filter-feeding ancestor to an actively ventilating predator capable of capturing larger prey. This early shift centered on the muscularization of the pharynx and the replacement of collagenous pharyngeal bars with more elastic cartilaginous elements capable of elastic recoil, rather than on the origin of jaws per se. According to this model, jaws arose only in a “final stage,” within the gnathostome lineage, whereas the “new head” evolved earlier and independently of jaws. Because jaws are a gnathostome innovation that appeared long after neural crest (including cranial cartilage) had evolved in the common ancestor of cyclostomes and gnathostomes, jaw evolution cannot, a priori, be invoked to explain the origin of the neural crest itself. Another central element of the “The New Head” concept was the elaboration of skeletal sensory capsules and associated support for expanding anterior neural structures. Northcutt (*34*) later clarified that what was proposed as “new” was an anterior prolongation of the alar central nervous system (CNS; forebrain) and the connective tissues mechanically creating and linking the sensory capsules. In this context, it is noteworthy that the olfactory and optic skeletal capsules are neural crest–derived, even in lampreys, whereas the otic capsule is mesodermal (*36*), which is strengthening the evolutionary connection between the crest and the most anterior components of sensory and nervous systems. Also, Gans and Northcutt (*27*, *33*, *35*) hypothesized that the earliest function of mineralization was related to facilitating the sensory capacity rather than purely protective: The high resistivity of hydroxyapatite in dentin and bone could enhance the performance of electrosensory lateral line organs, while dermal armor might shield these organs from extraneous signals and improve directional sensitivity. In line with a deeper sensory connection, Baker (*3*, *37*) noted that secretory calcium-binding phosphoprotein genes involved in biomineralization are also expressed in the developing and adult nervous system, where they contribute to glial functions in astrocytes.

**Fig. 1.**
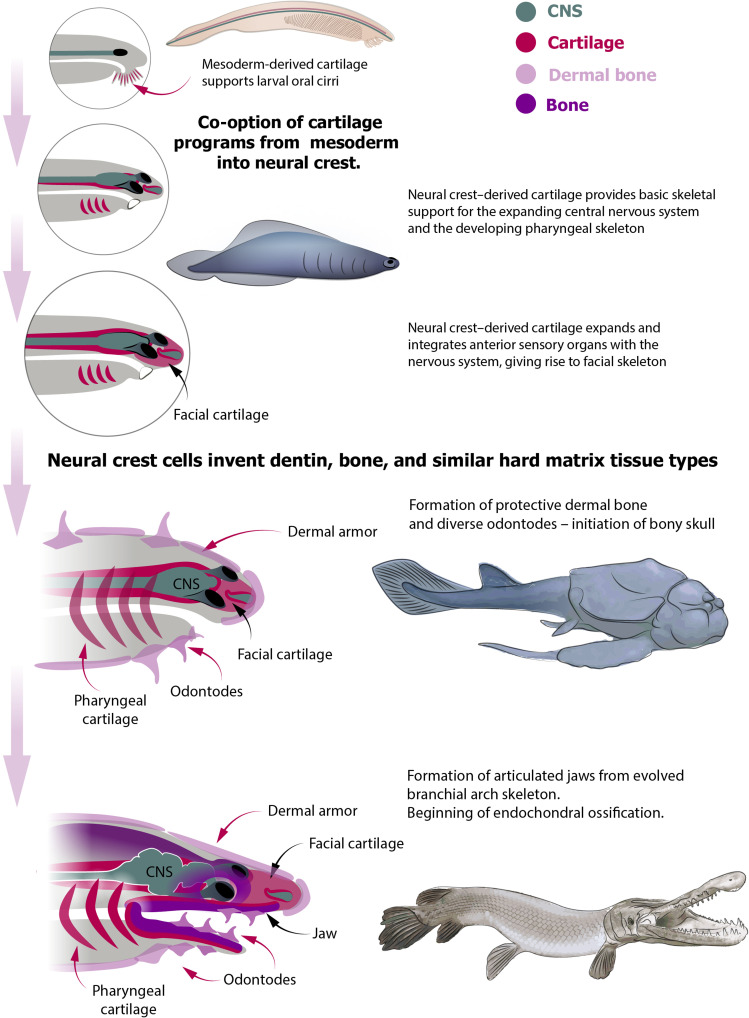
Suggested sequence of the evolution of the neural crest–derived skeleton. Note that the earliest benefits of the skeletogenic neural crest included support and mechanical integration of the CNS and sensory organs. Later, the innovation of bone and odontodes led to the formation of protective dermal bones and odontodes, lastly generating the skull and teeth. Articulated jaws came onto the scene very late in evolution. Artist Credit: O. Kharchenko, private biomedical illustrator, used with permission.

To summarize, the anterior body region of prevertebrate chordates was increasingly occupied by complex neural and sensory structures as body size increased and predatory behaviors emerged. Yet, those structures were lacking the internal skeletal support—possibly, there was not enough plastic mesoderm anterior to the notochord to provide it. The emergence of the cartilaginous skull—chondrocranium—was necessary to encapsulate fragile sensory organs and the increasing anterior end of the neural tube—future forebrain, also providing potential for the further expansion of the CNS (Fig. 1) (*38*). An analogous situation is seen in cephalopods, which develop supporting cartilages for the centralized parts of their nervous system and eyes (*39*). In another example, enlarged avian and reptilian eyeballs develop internal scleral bones, providing extra support to this key sensory organ (*40*). It seems that the centralized nervous system and sensory structures are sensitive to mechanical perturbations and physical damage, requiring extra support and shielding (*41*). In line with this hypothesis, the extinct jawless osteostracan *Norselaspis glacialis*—a representative of a sister group to gnathostomes—already exhibited skeletal elaborations associated with sensory organs (*42*). More broadly, the fossil record of jawless vertebrates reveals a substantial diversity of expanded cranial skeletal structures that protected and supported the CNS and sensory apparatus (*43*, *44*). These flexible and evolutionarily labile anterior skeletal regions were most likely produced by neural crest–derived ectomesenchyme, much as in modern lampreys and hagfish (*45*, *46*).

Overall, paleontological evidence shows that jawless chordates evolved an extraordinary range of skull morphologies and feeding strategies—some as intricate as the conodont feeding apparatus—long before the appearance of articulated jaws (*44*, *47*). This included the development of bone- and dentin-like tissues forming odontodes and heavy dermal armor, as seen in Paleozoic armored agnathans (*44*, *48*, *49*). One may also ask whether the neural crest–derived components of the bony skull, in addition to providing protection and structural support, might have played an early role in transmitting sound vibrations to the inner ear before the evolution of dedicated middle ear ossicles. In such a scenario, cranial dermal elements could have contributed not only to shielding and supporting sensory structures but also to mechanically coupling external stimuli to the inner ear, with specialized ossicles subsequently refining and taking over this function during later vertebrate evolution. Such innovations suggest that early neural crest cells played a pivotal role in the coevolution of the CNS, sensory systems, feeding apparatuses, and protective cranial structures before the emergence of gnathostomes. As Gans and Northcutt mentioned (*27*, *34*), the articulated jaw appeared much later, building upon capacities already established in neural crest–derived ectomesenchyme, which had acquired mesoderm-like skeletogenic potential (Fig. 1). In the following evolutionary epochs, neural crest also started to contribute to more complex thyroid (*50*) and advanced cardiovascular architectures (*51*, *52*).

It is also worth emphasizing that the cartilaginous skeleton surrounding the inner ear in fish is typically a mosaic of mesoderm- and neural crest–derived elements, indicating that neural crest is not strictly required to generate this type of skeletal support. The same principle applies to several other components of the cranial skeleton, including parts of the calvaria, the middle ear stapes, and the branchial cartilages of skates, where mesodermal contributions are substantial and, in some cases, predominant. In this light, it seems more accurate to view cranial neural crest ectomesenchyme as augmenting and extending the pool of mesenchymal cells available for head skeleton formation, rather than as a uniquely capable source of skeletal tissue. Its evolutionary role may have been to supplement, rather than replace, the mesenchyme provided by cranial paraxial mesoderm, thereby expanding the developmental and morphological potential of the skeleton supporting sensory organs and the rest of the head.

Moreover, although we originally linked the emergence of the neural crest to the mechanical protection of sensory cells with a focus on cranial structures, the available data suggest that this protective role was a property of the entire neural crest population including trunk, not just the cranial neural crest. Many stem gnathostomes had extensive dermal armor that covered large portions of the body, rather than being confined to the head. Consistent with this, recent work has shown that the trunk neural crest contributes to the postcranial dermoskeleton (*53*–*55*). Although this does not rule out the possibility that such contributions evolved convergently in some lineages, it is noteworthy that sturgeon scales derive from ancestral fish scales that already contained neural crest–derived innovations such as dentin-like matrices and bone. Moreover, dermal odontodes were not limited to the cranial region but also occurred postcranially in stem jawed vertebrates. Together, these observations support the view that the “ancestral” neural crest had odonto-skeletogenic potential along the entire body axis, enabling the evolution of “hard” protective tissues for sensory structures in both the head and trunk.

We also wish to more broadly consider the evidence that trunk neural crest cells in tetrapods retain the capacity to generate ectomesenchymal derivatives, including cartilage—albeit to a much more limited extent than cranial neural crest cells—across different groups such as amphibians and amniotes. For example, in frogs, the trunk neural crest normally contributes mesenchymal cells to the dorsal and ventral fins, as shown by interspecific trunk neural fold grafts (*Xenopus borealis* to *Xenopus laevis*) (*56*), as well as by using fluorescent dextran-labeled trunk neural folds and 1,1′-dioctadecyl-3,3,3′,3′-tetramethylindocarbocyanine perchlorate (DiI) labeling in *X. laevis* (*57*). In urodeles, interspecific trunk neural fold grafts between axolotl and triton revealed that trunk crest cells form connective tissue in the fins and contribute to the meninges (pia and arachnoid) (*58*, *59*). In axolotl, heterotopic grafts of trunk neural folds into cranial neural folds, followed by DiI labeling, demonstrated a small but clear contribution of trunk crest to branchial arch cartilages, indicating an intrinsic chondrogenic potential (*60*). By contrast, evidence for trunk neural crest contribution to the dermal bones of the turtle plastron remains rather indirect. Clark *et al.* (*61*) reported HNK1 immunoreactivity in plastron bones (*61*), but this marker alone does not establish lineage, and subsequent DiI-labeling studies showed a late wave of trunk neural crest migration into relevant regions without being able to follow these cells long enough to confirm their incorporation into the bones (*62*–*64*). At the same time, Yntema (*65*) did find that extirpation of the dorsal neural tube and adjacent trunk neural crest led to carapace defects alongside neural deficiencies, but overall, the trunk neural crest cell contribution to turtle plastron bones remains not fully clear. Next, in vitro clonal analyses of quail trunk crest show competence to form cartilage, bone, adipocytes, and smooth muscle cells (*66*, *67*). When loosely packed quail trunk neural crest cells were grafted into chick maxillary and mandibular primordia, they made a scattered but consistent contribution to chondrocytes in Meckel’s and scleral cartilage (*68*). Avian trunk neural crest cells can also be stably reprogrammed toward a more cranial-like state by forced expression of a cranial “subcircuit” (*Sox8*, *Tfap2b*, and *Ets1*). This reprogramming alters both their developmental potential in the native trunk environment—allowing them to generate vertebral cartilage—and their underlying gene regulatory program (*69*). Last, clonal cultures of the rat trunk neural crest readily yield smooth muscle actin–positive cells (smooth muscle cells or fibroblasts) in the presence of BMP2 (bone morphogenetic protein 2) or TGF-β (transforming growth factor–β) (*70*, *71*). The mouse trunk neural crest from thoracic-level neural folds was capable of forming teeth in explant coculture with mandibular epithelium (*72*) and generate cartilage nodules after culture with FGF2 (fibroblast growth factor 2) (*73*). The murine trunk neural crest also gives rise to a specific and stable population of mesenchymal cells—the endoneural fibroblasts, which support the endoneural extracellular matrix and facilitate nerve regeneration (*74*).

Despite these important observations showing that the trunk neural crest retains a limited capacity to generate mesenchymal and even skeletogenic derivatives, there remains a marked contrast with cranial neural crest, particularly in the robust production of skeletal tissues such as dermal bone and dentin. Thus, it is plausible that over the course of vertebrate evolution, the skeletogenic and odontogenic competence of the neural crest, which may have been present along the entire body axis initially, became progressively reduced and greatly restricted to the cranial region to enable better agility during the active movement. This appears as a consistent evolutionary trait, as comparative fossil and phylogenetic evidence indicates that early vertebrates commonly had extensive, often full-body dermal armor, whereas many descendant lineages show a recurrent, and in some cases progressive, reduction or complete loss of these bony coverings. This trend is well documented in the transition from heavily armored early jawless and jawed fish to modern actinopterygians with reduced or absent scales and in multiple archosaur and squamate clades where osteoderm loss is associated with more agile or specialized locomotor modes (*75*–*77*). It is further tempting to speculate that the molecular adaptations and gene expression programs behind the dermal bone, which underlie key neural crest–derived innovations, were later co-opted into the cartilaginous program, ultimately contributing to the origin of endochondral ossification and the ossified axial and appendicular skeleton.

Together, this supports a broader view of how the neural crest integrated into the existing germ-layer framework. As the neural crest emerged in early vertebrates as a young and nonobligatory lineage, it layered upon the already ancient and indispensable germ layer framework of ectoderm, mesoderm, and endoderm. In this context, the co-option of mesodermal mesenchymal programs into the neural crest did not replace an essential developmental role but instead duplicated it—generating a surplus of mesenchymal potential in a territory (the anterior ectoderm) where mesoderm was sparse or absent. This redundancy—a second mesenchymal system, derived from ectoderm—gave the organism a safe space for morphological experimentation in the most anterior domain. At the same time, the original mesodermal mesenchyme remained under strict regulatory control, responsible for building the somites, axial skeleton, cardiovascular system, and core musculature—features whose perturbation typically results in lethality. The neural crest, by contrast, was developmentally dispensable in early stages, producing accessory structures (supporting the evolving brain) that, while important, did not compromise viability if altered. This made the neural crest a low-risk, high-reward platform for evolutionary innovation. Also, the neural crest cells could execute chondrogenic programs outside the influence of mesodermal segmentation, Hox patterning, or notochord-derived axial cues. Over time, such decoupled mesenchyme underwent divergent evolution, giving rise to increasingly novel structures such as movable jaws, middle ear ossicles, horns, beaks, odontodes, and dermal armor, as well as various snout morphologies.

When discussing the innovation of cell types among mesenchymal derivatives of the neural crest, it is essential to clearly distinguish between cell types co-opted from the mesoderm—such as perivascular cells, smooth muscle, adipocytes, and dermal fibroblasts—and those that may represent true neural crest elaborations. For example, the larval amphioxus develops cartilage within its oral cirri to support the feeding apparatus (*78*), and vertebrate-like cartilage is also found in multiple protostome groups, including cephalopod mollusks and arthropods (*39*, *79*). This broad phylogenetic distribution indicates that the core genetic program for cartilage predates the origin of the neural crest and was almost certainly co-opted from mesodermal sources (Fig. 1) (*78*). The situation is more complex for bone and dentin. These mineralized tissues might appear to be genuine neural crest innovations (*54*), as their first appearance in the fossil record coincides with the emergence of the neural crest itself, and they are localized to the most anterior body regions (*48*).

Whether bone originated entirely within the crest or through contributions from mesoderm remains an open question. Nonetheless, well before the advent of articulated jaws, the cranial neural crest had already evolved the capacity to generate skeletogenic ectomesenchyme capable of differentiating into cartilage, dermal bone, and diverse mineralized tissues within odontodes (Fig. 1) (*44*, *48*, *49*, *78*). This is supported both by fossil evidence and by comparative embryology in extant jawless vertebrates, such as lampreys and hagfish, which lack jaws but still produce crest-derived skeletogenic tissues in the head (*38*, *44*, *80*). In this interpretation, the bone and the other mineralized matrixes were possibly innovated by the neural crest and then were secondarily co-opted into mesodermal derivatives. Modern dentin is produced exclusively by neural crest–derived cells, which strongly supports the idea that the neural crest played a central role in the innovation of dermal bone and dentin-like hard tissues. The fossil record also reveals numerous intermediate mineralized tissue types that form a continuum between modern dentin and bone(*48*, *81*), suggesting that these extinct and modern tissues are closely related and may even represent a “monophyletic” family of skeletal matrices. This view is further compatible with the notion that dermal bone first arose within ancient odontodes, where it would have served to anchor these units within soft dermal tissues and to structurally link them to one another.

## NEURAL CREST–DERIVED SOMATOSENSORY NEURONS EMERGED BY CO-OPTION OF ANCIENT PROGRAMS

In vertebrate animals, multipotent neural crest cells give rise to somatosensory neurons—specialized cells responsible for detecting a wide range of stimuli such as touch, pain, temperature, and proprioception—the sense of body part positioning and movement (*82*, *83*). The majority of these neurons are organized into dorsal root ganglia (DRGs), located bilaterally along the spinal cord. DRG neurons are pseudounipolar, having a single process that bifurcates into two branches: one extending peripherally to innervate the skin, muscles, or internal organs and the other projecting centrally into the spinal cord to relay sensory information to interneurons and, ultimately, to the brain (*84*). This peripheral sensory system is essential for an animal’s ability to perceive and respond to its environment, supporting behaviors vital for survival, such as withdrawal from harmful stimuli, coordinated locomotion, and postural control.

**Fig. 2.**
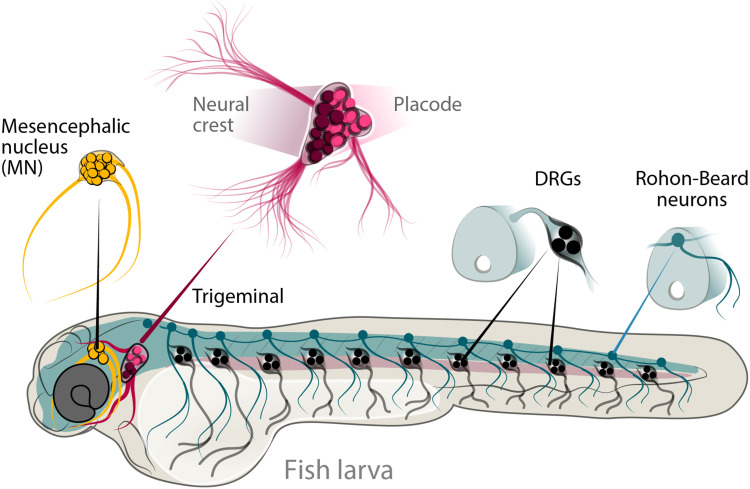
Four types of “parallel” somatosensory systems in anamniotes: neural crest–derived DRGs, trigeminal placode–derived neurons, Rohon-Beard sensory neurons, and proprioceptive neurons of mesencephalic trigeminal nucleus. Artist Credit: O. Kharchenko, private biomedical illustrator, used with permission.

However, it would be misleading to consider the peripheral sensory apparatus of vertebrates—particularly the DRG somatosensory system—as an entirely novel feature restricted to the neural crest derivatives (Fig. 2). While the neural crest plays a central role in forming the vertebrate-specific architecture of somatosensory pathways, the overall organization and function of these neurons echo more ancient systems. For instance, DRG neurons bear notable structural, transcriptional, and functional similarities to mechanosensory and proprioceptive neurons found inside of the vertebrate CNS, as well as in the nervous systems of protostome and deuterostome invertebrates. The vast majority of multicellular animals does have cells and neural circuits that detect touch and mechanical pressure and provide muscle feedback.

In particular, the Rohon-Beard neurons—dorsal spinal cord–residing sensory cells found in lampreys (*85*–*87*), jawed fish (*88*), and amphibians (*89*, *90*)—are functionally and molecularly similar to DRG neurons (*88*, *91*). Although Rohon-Beard cells arise from the dorsal neural tube rather than the crest, their sensory role and gene expression profiles overlap with neural crest–derived somatosensory neurons (*91*). Rohon-Beard neurons are situated just beneath the roof plate and within the dorsal-most region of the neural tube, often found at regular intervals. Their cell bodies are large, and they typically give rise to two main types of processes: a peripheral axon that extends to the skin and a central axon that projects within the spinal cord. Similar to DRG neurons, their peripheral axons usually branch extensively beneath the epidermis, forming a dense meshwork that enables detection of touch (*88*). In summary, Rohon-Beard neurons represent a developmentally ancient, spinal-cord intrinsic sensory neuron population with early embryonic origin, simple connectivity, and critical functional importance for initial sensory-motor behaviors in aquatic vertebrates.

Thus, in the trunk of fish and amphibians, there are two distinct and parallel somatosensory systems: One consists of Rohon-Beard neurons, which originate within the spinal cord, and the other is made up of DRG neurons derived from the neural crest (Fig. 2). The Rohon-Beard neurons are typically described as a temporary population that disappears early in development, with their functions taken over by the DRG neurons. However, recent studies revealed that they are still present from 1 day after fertilization up to at least 15 days when zebrafish have already entered the juvenile stage and are capable of complex behaviors (*92*–*94*). This shows that fish maintain both somatosensory systems (Rohon-Beard + DRGs) in parallel for a long time.

The second direct example of ancient non–neural crest–derived somatosensory neurons is found in the mesencephalic trigeminal nucleus, which houses proprioceptive neurons derived from the alar plate of the midbrain (Fig. 2) (*95*, *96*). These neurons serve the same role as TrkC+ proprioceptive DRG neurons derived from the neural crest. The mesencephalic trigeminal nucleus houses primary sensory neurons that monitor the stretch and tension of jaw muscles, including muscle spindles and mechanoreceptors in the ligament. To understand the evolutionary history and function of these cells, it is important to consider their role both before and after the origin of jaws and jaw muscles (*97*). In jawless vertebrates and early gnathostome ancestors, proprioceptive control of anterior somite-derived musculature (such as premandibular and branchial arch muscles) would have been critical for precise head movements, feeding mechanisms, and perhaps ventilation. In these ancient lineages, the midbrain-derived sensory neurons may have innervated cranial muscles used for opening the oral cavity, directing water flow, or stabilizing head posture—functions that would later be elaborated into the fine-tuned proprioceptive feedback required for active prey capture via jaws. This idea is contrary to the scenario previously suggested by Sato (*97*) and where articulated jaws played a leading role in stimulating the evolutionary origin of proprioceptive neurons of the mesencephalic trigeminal nucleus. Here, we develop a different idea: With the evolution of jaws in early gnathostomes, a transformation occurred in the craniofacial musculoskeletal system—muscles derived from the first pharyngeal arch gave rise to the jaw adductors, and novel muscular and skeletal arrangements enabled biting, grasping, and more complex manipulative behaviors. This evolutionary innovation placed new demands on proprioception to precisely coordinate force and position during feeding. The midbrain mesencephalic trigeminal nucleus neurons, already present and potentially associated with ancestral head muscles, were now able to serve this new functional context by providing rapid feedback for the regulation of jaw movements. In modern jawed vertebrates, the mesencephalic trigeminal nucleus remains critical for this role. It provides direct input to motor neurons in the brainstem, forming part of a reflex arc for jaw closing and bite force modulation (*98*, *99*). It seems that the mesencephalic trigeminal nucleus represents a conserved, non–neural crest–derived lineage of proprioceptors that was already present before the evolution of the neural crest and cranial sensory ganglia and was later retained and functionally integrated into the control of gnathostome jaw apparatus.

Last, placodally derived viscerosensory and somatosensory neurons, particularly those of the cranial sensory ganglia such as the trigeminal ganglion, also play a crucial role in conveying sensory information from the face and internal organs to the CNS. These neurons arise from the cranial neurogenic placodes, which are ectodermal thickenings in the embryonic head region that contribute to the formation of several cranial sensory structures. In the case of the trigeminal ganglion, which contains neurons responsible for facial somatosensation, its sensory neurons derive from three converging embryonic sources: the cranial neural crest and two neurogenic placodes—the Pax3-positive ophthalmic trigeminal placode (termed “profundal placode” in anamniotes) and the Pax3-negative maxillomandibular trigeminal placode (termed “trigeminal” placode in anamniotes) (*100*, *101*). Similarly, visceral sensory neurons—such as those in the nodose and petrosal ganglia, which relay information from internal organs (e.g., blood pressure and gut distension)—derive from the epibranchial placodes (*83*).

While these neurons come from different embryological origins (placodal ectoderm versus neural crest), they converge on highly similar molecular and functional programs. In the trigeminal ganglion, neural crest–derived and placodal neurons intermingle and often become indistinguishable on the basis of mature molecular profiles (Fig. 2) (*102*). Although some fate mapping studies show biases in what types of neurons each lineage gives rise to [e.g., placodal cells more often give rise to mechanoreceptors, while crest-derived neurons contribute more to nociceptors and proprioceptors (*103*)], there is considerable functional and molecular overlap (*101*, *104*). This shared genetic toolkit suggests that cranial placodal neurons and neural crest–derived peripheral neurons may have evolved through interrelated evolution.

At present, it remains unclear which preneural crest structure carries the most ancient somatosensory and viscerosensory programs—whether it is the placodal ectoderm or the CNS itself. However, across a wide range of metazoans—including hemichordates, basal chordates like amphioxus or tunicates, echinoderms, and even protostomes such as *Drosophila*—sensory neurons consistently arise from placode-like epithelial thickenings (*105*). In amphioxus and hemichordates, for instance, somatosensory neurons are derived from distributed epithelial microplacodes along the body surface (*106*, *107*), suggesting that placodal neurogenesis is both ancient and conserved. In tunicate *Ciona*, the sensory bipolar tail neurons (BTNs) resemble placodally derived sensory neurons of vertebrates and can be transformed into classic placodal derivatives—pulp sensory cells or even into the hybrid BTN-pulp sensory cell type (*108*). BTNs have a migratory progenitor and are responsible for somatosensation—features that also align them with neural crest–derived DRG neurons (*14*). However, this hypothesis is rejected in the latest studies because of the aforementioned reasons. Therefore, the ancestral migration of neuroblasts and their precursors is not a unique neural crest–specific feature (*4*, *100*, *109*–*111*).

From an evolutionary perspective, it is therefore possible that placodally derived somatosensory neurons represent the most ancestral mechanism of sensory neuron development. The centralization of the nervous system, culminating in the formation of the neural plate and its folding into the neural tube in vertebrates, may itself be interpreted as a secondary elaboration of this original placodal architecture—a massive, contiguous placode. In this view, the neural tube can be seen as an expanded epithelial placode where neurogenesis occurs in situ, and neurons manage to stay within the provided epithelial space without any need to migrate through the basal membrane into mesenchymal spaces (*112*). This is the major innovation in deuterostomes (a consequence of innovated radial glial cell architecture), whereas the neurogenic placodes or neurogenic epithelia, which generate neuroblasts migrating through the basal membrane into underlying mesenchyme to form coalescing ganglia, represent a solution dominating both protostome and deuterostome nervous systems. It is possible that the neural tube evolved by spatially extending and integrating ancient placodal neurogenic mechanisms within an innovated radial glial cell architecture (*112*).

Overall, we can conclude on the functional and gene expression convergences, where evolution has permitted multiple developmental pathways to produce the same functional cell phenotype from different developmental origins—a somatosensory primary neuron. Such redundancy may reflect the evolutionary duplication and redistribution of a fundamental somatosensory neuronal module, with the neural crest serving as a means to expand the spatial domain of this neuronal identity.

These suggest that the neural crest has an ability to incorporate and adapt preexisting neuronal programs, originally confined to central and non–neural crest sources and redeploy them in the neural crest context. Potentially, this adaptive reuse is made possible by the modular nature of the gene networks governing sensory neuron development, particularly involving transcription factors such as Brn3a (Pou4f1), Isl1, Neurog1/2 (neurogenin 1/2), and Runx1/3 (runt-related transcription factor 1/3), as well as neurotrophin receptors (TrkA/B/C—also known as Ntrk1/2/3) (*113*).

**Fig. 3.**
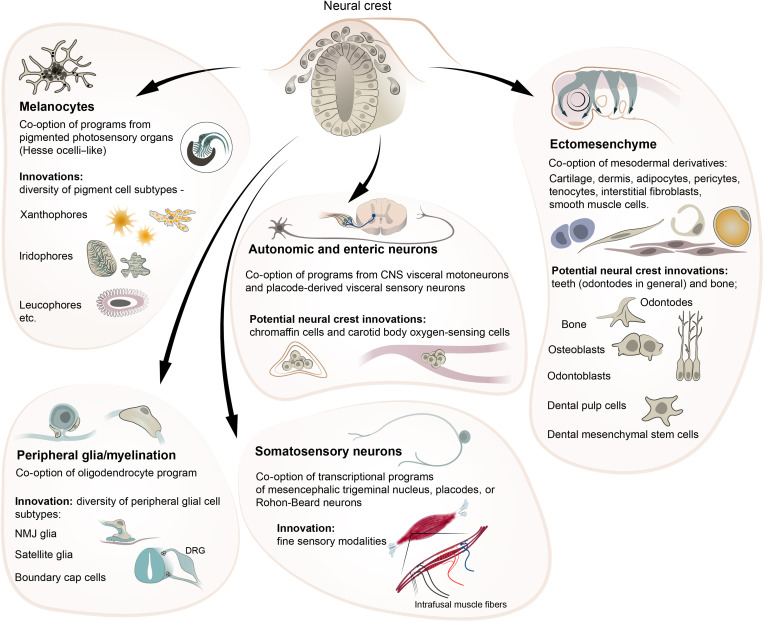
Suggested co-options and innovations in the evolving neural crest lineage. Artist Credit: O. Kharchenko, private biomedical illustrator, used with permission.

The co-option of the corresponding cell identity programs could occur at the time of the neural crest origin (or soon after) (*8*), although some finely adapted modalities and sensory cell subtypes could emerge within the neural crest lineage as a true innovation in a group-specific manner. For example, Usoskin *et al.* (*114*) revealed the incredible functional diversity of somatosensory neurons in mammals by profiling their mechanosensitivity in vitro at the single-cell level (*114*). The authors of the corresponding study used a high-throughput platform to record and classify more than 1800 sensory neurons from mouse DRG, allowing the construction of a detailed map of functional subtypes based on mechanical stimulus responses. The key finding was that somatosensory neurons comprise multiple functionally distinct subtypes with varying types and fine subtypes of mechanosensitivity (*114*). The resulting classification of neurons turned out to be based on parameters such as mechanical threshold, adaptation rate, and current amplitude, resulting in the identification of more than 10 functionally defined subpopulations. Furthermore, the study integrated electrophysiological properties with gene expression profiles, which suggested that developmental programs and molecular markers can be predictive of the fine sensory modality a neuron will adopt. This complexity of fine subtypes possibly evolved secondarily already in the neural crest lineage (Fig. 3) and represents a true marvel of sensory capacity—potentially going beyond the preneural crest systems in its fineness.

## AUTONOMIC AND ENTERIC NEURONS: CO-OPTION OF VISCERAL CNS CIRCUITS INTO THE NEURAL CREST

Neural crest cells contribute not only to somatosensory neurons but also to the formation of the secondary (postganglionic) neurons of the autonomic nervous system, including the residents of sympathetic, parasympathetic, and enteric ganglia (*32*, *115*–*119*). These peripheral autonomic neurons are essential for regulating the function of internal organs such as the heart, kidneys, gastrointestinal tract, and others. In the vertebrate nervous system, these peripheral ganglia are innervated by primary visceral motor neurons that reside within the CNS, particularly in the brainstem and spinal cord (*83*, *120*).

We find it plausible that peripheral autonomic neurons originated through evolutionary duplication and redeployment of central visceral motor circuits. Such evolutionary remodeling may have involved the co-option of core visceromotor transcriptional programs by neural crest cells, allowing them to generate novel neuronal identities within the peripheral nervous system. This hypothesis is supported by the molecular similarity between central visceral motor neurons and peripheral postganglionic autonomic neurons. Visceral motor neurons in the brainstem, and the postganglionic neurons they connect to in peripheral ganglia, depend on a common set of transcription factors, most notably Phox2a (paired like homeobox 2A) and Phox2b. These factors are not used by somatic motor neuron lineages (*121*). Even viscerosensory neurons that come from placodes and send signals from internal organs back to the brain also rely on Phox2a/b despite forming different central circuits from motor neurons (*122*, *123*). For viscerosensory neurons, particularly those in the nodose ganglion, the Phox2b transcription factor plays a pivotal role, highlighting a shared genetic program for visceral navigation and innervation, regardless of developmental origin (*122*), which is further supported by the fact that proneurogenic Schwann cell precursors (SCPs) residing in visceral nerves also express Phox2b, whereas the motor nerve–associated or skin sensory SCPs do not express it (*116*, *118*).

Comparative data from basal chordates and invertebrates further support this evolutionary conservation. In these nonvertebrate deuterostomes, visceral motor neurons project directly from the CNS to the peripheral effector tissues without intervening ganglia, indicating a simpler ancestral organization. Similarly, in protostomes such as mollusks, visceral motor neurons express homologs of Phox2a/b and send direct projections to target tissues (*121*). These facts suggest that the ancestral deuterostomes including prevertebrate chordates had a unified Phox2-dependent visceral motor circuit located entirely within the CNS. The vertebrate condition appears to have emerged through a hypothetical duplication of this central motor program, with one copy remaining in the CNS and the other being redeployed into the newly evolved neural crest lineage. The neural crest, with its capacity to incorporate a wide range of cell types, served as a developmental conduit through which this ancient motor circuitry was reexpressed in novel anatomical contexts outside of the CNS (Fig. 3). This event enabled the formation of peripheral autonomic ganglia and the enteric nervous system as distinct anatomical entities.

An alternative interpretation is that neural crest cells are essentially CNS-like progenitors that have migrated into the periphery and thus retain the ability to generate the same major neuronal classes as in the CNS, including Phox2b-dependent motoneurons. Neural crest cells can be viewed as CNS-born progenitors with a general capacity to produce CNS-like neurons, as exemplified by Rohon-Beard–like sensory neurons in DRGs. However, this raises a specific difficulty: In the CNS, motor neurons are ventral, whereas neural crest originates dorsally, and dorsal neural progenitors are more naturally aligned with sensory rather than motor lineages (*124*). This topological mismatch makes it more plausible that autonomic motor programs were secondarily co-opted from ventral CNS circuits into the dorsally derived neural crest lineage, rather than representing an intrinsic ancestral property of dorsal progenitors. Such co-option could have been facilitated by the broadly permissive, CNS-like chromatin and regulatory landscape of neural crest cells, which may have allowed ventral motor programs to be redeployed in a peripheral context.

At later evolutionary stages, the subtypes of sympathetic, parasympathetic, and especially enteric neurons diversified into a large number of specialized modalities including neuroendocrine chromaffin cells (*125*) and also a multitude of enteric interneuron and sensory subtypes (*126*, *127*), which overall represents a true innovation of the neural crest lineage. Among other improvements, this diversity in the evolving gastrointestinal system could essentially boost the cross-talk between the nervous system of a gut with microbiota and immune cells, possibly inventing the vertebrate “gut-brain axis” (*128*) together with the nodose placode–derived sensory neurons (i.e., vagal afferents), which provide a direct connection between the gut and the hindbrain (via the vagal nerve) (*129*).

## PERIPHERAL GLIAL CELLS OF VERTEBRATES: WHERE DO THEY ARISE IN EVOLUTION

The current repertoire of embryonic and adult peripheral glial cells (including myelinating, nonmyelinating, and terminal Schwann cells; SCPs; boundary cap stem cells; and immature glial cells) suggests similarities between myelination programs in CNS oligodendrocytes and peripheral myelinating Schwann cells (*130*). However, it is hard to reason about the source of potential innovation of myelination, as it arose already in vertebrates having the neural crest. The extant cyclostomes (jawless fish) do not show signs of myelination, although their ion channels start to show some degree of clustering, facilitating saltatory propagation of the action potential (*131*). It might be that the myelination emerged in the brain to enable the increase in the brain size and the speed of information processing, or alternatively, it could have been acquired as the result of the peripheral body extension and the need to send and receive fast-propagating stimuli to and from the muscles and skin. Overall, current knowledge about the evolution of myelination in the nervous system offers little insight into whether this trait was co-opted into or out of the neural crest lineage and thus does not significantly clarify the mechanisms behind the expansion of neural crest fate potential. In contrast, another member of the glial lineage—the SCP—stands out as particularly intriguing from an evolutionary perspective (*132*, *133*).

One of the most notable features of SCPs is their multipotent differentiation potential similar to their maternal population—the neural crest. In modern vertebrates, they can give rise to melanocytes; parasympathetic, sympathetic, and enteric neurons; chromaffin cells of the adrenal medulla; dental pulp cells and odontoblasts; endoneurial fibroblasts; and occasionally, to cartilage and bone of a developing head, ribs, and the scapula (*133*, *134*). Often, SCPs contribute to derivatives in late-emerging structures, such as parasympathetic ganglia (*115*, *135*), adrenal medulla (*125*), or developing teeth (*136*), long after the migratory neural crest disappears in an embryo. This is a highly conserved feature, because in lampreys, SCPs contribute to the trunk enteric nervous system (*137*). This capacity is not merely a retained plasticity but appears to be an active, regulated multipotency embedded within their transcriptional program, which is highly similar to the migratory neural crest (*118*). In a way, SCPs represent a nerve-associated phase of the neural crest multipotent state and shall be formally defined as nerve-associated and biased neural crest cells.

Evolutionarily, this suggests that SCP-like cells could have served as “mobile intermediates,” capable of delivering versatile progenitors to different regions of the body, where local cues could then determine their final identity. This would have permitted early vertebrates to deploy peripheral neural, pigment, skeletal, and endocrine cells without requiring independent migratory mechanisms for each lineage. In extant vertebrates, SCPs are known to travel with peripheral nerves, colonizing distal regions of the body during development. This nerve-associated movement provides a guided migratory route, allowing SCPs to reach peripheral targets without the need for full intrinsic motility or pathfinding capacity (*133*). This feature may echo an ancestral strategy in early vertebrates or prevertebrate chordates, where protoneural crest cells lacked the autonomous EMT-based migratory program but could still spread along axons of nascent sensory or motor neurons.

In the following phase, the evolution of free migratory neural crest cells, capable of delaminating from the dorsal neural tube and moving along mesodermal substrates, required a suite of additional innovations—down-regulation of epithelial adhesion molecules (cadherins), up-regulation of EMT drivers (Snail and Twist), and the emergence of directional motility machinery (*21*). SCPs, in this context, may represent an evolutionary ancient adaptation: cells that were migratory in a tethered, scaffolded manner while progressively acquiring the transcriptional tools necessary for autonomous migration. In other words, axonal routes may have provided an evolutionary “training ground” for the eventual development of independent cell migration. This strategy would have been efficient, low-risk, and allowed for modular acquisition of new migration-associated functions. In this light, the neural crest did not begin as a migratory supercell but evolved through incremental acquisition of dispersion and plasticity, scaffolded by interactions with the nascent peripheral nervous system.

## MELANOCYTES CONNECT TO THE VISUAL SYSTEM: PARALLELS WITH THE RETINAL PIGMENTED EPITHELIUM

Last, we turn to the evolutionarily significant case of melanocytes—photosensory, pigmented cells derived from the migratory neural crest and SCPs (*132*, *138*). In vertebrates, melanocytes are not merely pigment producers but dynamic effectors capable of responding to visible and ultraviolet (UV) light (*139*, *140*). For instance, Shiraki and colleagues (*140*) showed that early zebrafish larvae respond to light with rapid melanosome dispersion within their pigment cells and that this response is mediated by extraocular, melanophore-intrinsic photoreception. Eye enucleation does not abolish this light-induced dispersion, indicating that melanophores themselves autonomously sense light and actively redistribute their pigment granules. A simple nocturnal observation in amphibians and fish further highlights this autonomy: When a flashlight beam is directed onto the skin of a frog or fish in complete darkness, the melanophores (often referred to as melanocytes) rapidly contract, pulling their melanosomes toward the cell center. Within less than a minute, the cells appear as small dark dots, and the overall skin tone becomes visibly lighter. When the light is removed and the animal returns to darkness, the melanosomes gradually redistribute throughout the dendritic processes within the melanophores, darkening the skin once again. This dynamic response works in isolated and cultured melanophores as well (*139*). This reversible pigment migration provides an effective demonstration of the photosensitive and camouflaging capabilities of these cells. However, despite all this evidence, the photosensory function of melanocytes and their similarity to pigmented ocelli are rather ignored, even though these pigmented cells express melanopsins and components of the phototransduction cascade (*8*).

An additional, and evolutionarily critical, site of melanin-based pigmentation is found in the vertebrate eye—specifically, in the retinal pigmented epithelium (RPE) (*141*). This layer of darkly pigmented cells lies immediately posterior to the retina and serves several key functions, including the absorption of stray light, metabolic support of photoreceptors, and the maintenance of visual acuity. RPE cells express a suite of transcription factors and enzymes critical for melanin biosynthesis—SOX10, MITF, TYR (tyrosinase), TYRP1 (tyrosinase-related protein 1), and DCT (dopachrome tautomerase)—mirroring the molecular program seen in melanocytes (*24*). Yet, the embryonic origin of the RPE is neuroepithelial: It arises from the optic cup, not the neural crest (*141*). This presents a developmental paradox: two distinct lineages, neural crest and neuroepithelium, converging on a markedly similar gene regulatory network and organelle specialization (melanosomes) to produce functionally analogous, pigmented, photosensitive cells.

This convergence raises a fundamental evolutionary question: Do melanocytes and RPE cells share a deep homology, or did they arise because of convergent co-option of a pigmentation program? The recent transcriptomic and epigenomic study suggested the former (*24*). When examining gene expression profiles and chromatin accessibility landscapes beyond pigmentation genes, we have found an unexpectedly high degree of similarity between melanocytes and RPE cells. These data support the hypothesis that both cell types derive from a common ancestral pigment cell—resembling the pigmented photoreceptors of protochordates, such as the ocellus of *Ciona* or the Hesse ocelli of amphioxus (*24*). These ancient cells combined melanin-based optical shielding with light detection, a functional pairing that remains tightly linked across metazoan evolution (*8*). For instance, cubozoan jellyfish have camera-like eyes with pigmented cups that function as directional light sensors (*142*), while even pinealocytes of the mammalian pineal gland—descendants of photoreceptors—retain the capacity to produce melanin (*143*).

**Fig. 4.**
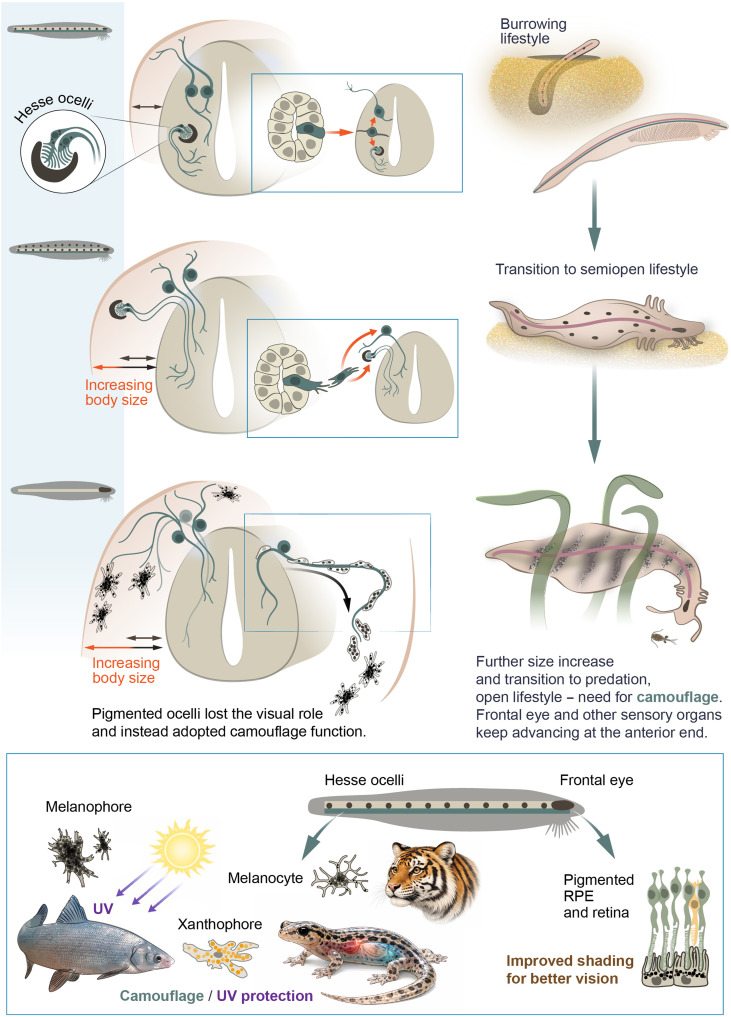
Suggested evolutionary scenario for the origin of nascent neural crest cells. First, being driven by the animal size increase, the intra-CNS pigmented ocelli, which are dispersed throughout the length of the neural tube, translocate toward the body periphery via emigrating progenitors. As the body size continued to expand and predatory behavior evolved, the visual function of these subcutaneous pigmented ocelli became obsolete (previously, it has been associated with the burrowing behavior). Instead, these peripheral pigment cells were repurposed for adaptive coloration, giving rise to melanophores capable of dynamic light–dependent camouflage. Over time, migratory melanophore progenitors, representing a nonessential and evolutionarily flexible lineage, became a substrate for further fate diversification through co-option of additional developmental programs—marking the onset of the multipotent neural crest. Artist Credit: O. Kharchenko, private biomedical illustrator, used with permission.

This evolutionary scenario suggests that the protoneural crest did not invent melanocytes de novo, but instead, they inherited a deeply integrated pigment-photoreceptor program by lineage (see Fig. 4) (*8*, *24*). In doing so, the neural crest not only preserved the ancient linkage between melanin and light perception but also expanded its functional scope. One of the key innovations in vertebrate melanocyte biology is the ability to transfer melanosomes to neighboring keratinocytes in the integument (*144*). This feature, absent in RPE cells or simple photoreceptors, enables pigmentation of hair, feathers, scales, and skin and has contributed to the evolution of complex signaling displays, camouflage mechanisms, and enhanced protection against UV radiation.

Moreover, vertebrate pigment cells have undergone extensive diversification. In addition to melanocytes, other neural crest–derived chromatophores—such as xanthophores, erythrophores, iridophores, and leucophores—confer a broad palette of colors and optical effects (*145*–*148*). These cells use mechanisms ranging from pigment synthesis (e.g., carotenoids and pteridines) to structural coloration via reflective crystals (e.g., guanine platelets) (*147*). The diversification of pigment cell types exemplifies how the neural crest, through its inherent plasticity and capacity for developmental innovation, has harnessed ancestral programs and transformed them into novel adaptive features that shape the organismal appearance and function.

## CAMOUFLAGE-CENTRIC HYPOTHESIS OF THE NEURAL CREST ORIGIN DURING TRANSITION TO PREDATION

What was the “Old Head” in comparison to the “New Head” proposed by Gans and Northcutt (*27*)? To understand this distinction, we can explore the extant neural crest–lacking deuterostomes such as amphioxus, hemichordates, and tunicate larvae. These animals already display anteriorly localized structures such as skeletal or supporting elements, complex feeding apparatuses, and basic sensory organs—all contributing to a functional “old face” (*38*, *42*, *43*). Despite lacking neural crest cells, these organisms have a preexisting head region with critical capabilities. This indicates that the most primary neural crest did not give rise to the head de novo but instead emerged within an already functional anterior framework. The “New Head,” as defined in vertebrates, took shape over a prolonged evolutionary timescale and did not emerge immediately in the earliest protoneural crest–bearing animals.

This brings us to a fundamental question: If not for the complex structures of the modern vertebrate face, what was the original evolutionary driver for the emergence of the neural crest? We must avoid assuming retroactive logic, where the benefits of later complexity are projected onto early stages. Instead, the initial function of the neural crest must have provided a near-immediate adaptive advantage—something simple, yet beneficial enough to be positively selected and retained. Only later would this lineage expand in potency and versatility, incorporating mesodermal features and contributing to the remodeling of the face and head into a more flexible and modular structure.

It is unlikely that duplications of somatosensory (*5*, *85*) or visceromotor systems were under the original selective pressure driving neural crest emergence. Similarly, it is hard to argue that the transformation of existing peripheral glial cells [discussed in (*3*)] alone would provide a strong enough advantage—especially if peripheral glia already existed in ancestral chordates. So, what then did ancestral chordates lack, and what function could neural crest–derived cells uniquely provide?

A compelling hypothesis points toward a fundamental, survival-critical function: protection from UV radiation and dynamic camouflage [(*6*), chapter 5; (*8*, *24*)]. The invertebrate chordate amphioxus, which leads a benthic, sediment-burrowing lifestyle, is largely translucent and lacks widespread body pigmentation; melanin is confined to localized photosensory structures in the CNS, such as the Hesse ocelli distributed along the length of the neural tube—features that are advantageous for controlling burrowing and hiding behavior. If the ancestral chordate is assumed to have resembled amphioxus, then the subsequent transition in some lineages from passive filter-feeding to active swimming and predation in the open water column would have made melanin-based camouflage and UV protection a critical adaptation.

To meet this challenge, early chordates may have repurposed noncritical pigmented photoreceptors—similar to those found in their CNS-based visual systems—and turned them into melanin-producing, photosensitive cells in the peripheral skin (Fig. 4) (*8*). These migrating photosensory pigment cells, precursors to modern melanocytes or melanophores, would constitute the earliest neural crest derivatives. Such cells would have required CNS-like progenitors capable of migrating out of the neural tube—a novel but minimal innovation that offered immediate benefits. The migrating nervous system progenitors (for instance, BTNs) are found already in *Ciona*, where they give rise to some neuronal subtypes (*14*). Also, in larger animals, such migration could have been guided by existing peripheral nerves, as seen in modern SCPs (*133*), which are nerve-associated, multipotent, and migratory and also form melanocytes in normal development. In line with this logic, SCPs could originate from the cells that resemble intra-CNS migratory progenitors, which are known to migrate along the neuronal processes or extended radial glial cells (*110*, *111*, *149*).

Thus, the original function of the neural crest may have been the deployment of CNS-derived, pigmented photosensitive cells to the periphery for camouflage and UV defense. This photosensory pigmentation system may represent the evolutionary kernel around which the broader neural crest gene regulatory network later crystallized. The earliest lineage of photosensory, pigmented peripheral progenitors was possibly flexible enough to incorporate or “access” additional cell fates—an adaptability made possible by the fact that variations in pigmentation, while potentially subject to natural selection, are not lethal or severely damaging. This relative nonessentiality and developmental plasticity would have made such early protoneural crest cells especially suitable as a substrate for evolutionary experimentation. Initially restricted to one or two terminal cell types, this pigment-producing lineage may have gradually expanded its potential by co-opting additional transcriptional modules for multipotency, nerve-independent migration, and differentiation into diverse derivatives—including craniofacial skeleton, peripheral neurons, and glia. Over time, this expansion enabled the formation of the vertebrate “New Head” (*27*). The example of such expanded potential includes the recent finding of *Ciona*’s pigment lineage progenitors giving rise to parts of nervous system after metamorphosis. These a9.49 progenitor cells localize to the edges of developing sensory vesicle—a location conceptually similar to the position of the cranial neural crest—and give rise not only to the pigmented ocellus but also to the later nervous system derivatives (*150*). On a different note, a recent single-cell transcriptomics study suggests the presence of a cell population in amphioxus larvae that may resemble migratory neural crest cells and is hypothesized to delaminate from the anterior neural tube and migrate ventrally (*151*). The genes expressed in the corresponding migratory neural crest cell–like cluster include MITF—a key melanocyte-specifying transcription factor. However, at present, however, further work is required to demonstrate unambiguously both the multipotency and migratory behavior of these cells before they can be reliably regarded as homologous to the vertebrate neural crest. Still, in light of the abundance of pigmented photosensory organs at the anterior end of amphioxus, this observation points to an interesting future research avenue: Forthcoming lineage-tracing experiments will be able to test whether this putative neural crest–like population is truly related to pigmented photoreceptors and thus may either support or refute the central hypothesis proposed here.

An alternative hypothesis to the one homologizing photosensory organs and melanocytes would be that ancient peripheral glial cells incorporated a co-opted pigmentation module. This is also plausible because, in adult amphioxus, axon-ensheathing peripheral glial cells have been described along the dorsal roots and their peripheral branches. Early light-microscopic studies identified these “Müller’s glia” as small cells with intensely stained nuclei clustered around dorsal nerves and their branches and between muscle tails (“false ventral roots”), where they lack neurites but can form partial sheaths around axons and muscle tails (*152*, *153*) Subsequent electron microscopy by Peters (*154*) confirmed that the cells associated with dorsal roots are axon-ensheathing glia: Their “sheath cells” extend thin cytoplasmic sheets along the nerves, closely resembling the way Schwann cells surround unmyelinated axons in vertebrates. These amphioxus glial cells, briefly mentioned as “Müller’s glia” in the review by Wicht and Lacalli (*155*), thus demonstrate the presence of peripheral axon-ensheathing glia in extant invertebrate chordates, even though their developmental origin remains unknown. Therefore, resolving their origin is a key for understanding the evolution of the neural crest. The idea that Müller’s glia-like cells were the initial and primary fate of the earliest neural crest population is neither supported nor definitively excluded by current molecular data, which instead emphasize extensive transcriptional and functional similarities (beyond the pigmentation module) between melanocytes and other photosensory cell types such as the retinal pigment epithelium, photoreceptors, and pinealocytes (*24*). These comparative data suggest that melanocytes did not arise by co-option of pigmentation into glial cells from visual pigmented organs but rather originated directly from the lineage of CNS-residing pigmented photoreceptors. Being carried out of the CNS during the neural crest origin, pigmented photosensory cells kept an uninterrupted evolutionary connection with the main visual systems, together representing the cases of homologous cell types. This exit from the CNS could occur via nerve-associated migration of neuroglial progenitors of pigmented ocelli while gradually acquiring multipotency and giving rise to the modern neural crest and SCPs.

## TRANSFORMATIVE POTENTIAL OF THE NEURAL CREST

The evolution of the neural crest is not merely a story of adding new cell types or building large flexible morphologies—it may have begun with the emergence of a single, immediately beneficial function: providing camouflage and UV protection through pigmented, photosensitive cells. From this modest beginning, the neural crest stretched into something far more transformative: a lineage that expands the morphogenetic toolkit without jeopardizing existing systems. In this sense, the neural crest represents a higher-order evolutionary innovation—not a trait but a platform for traits. It became a workshop where old parts were refitted into new structures: Somatosensory and secondary visceromotor neurons were co-opted from ancestral placodal or CNS programs, while cartilage-forming capabilities were recruited from mesodermal origins. In its later evolutionary phases, the neural crest functioned as an evolutionary sandbox—a permissive environment for testing and assembling new traits atop previously co-opted identities—in contrast to the more conserved, constrained mesoderm—allowing vertebrates to engage in rapid morphological and functional experimentation.
